# Toxicity of Thallium at Low Doses: A Review

**DOI:** 10.3390/ijerph16234732

**Published:** 2019-11-27

**Authors:** Beatrice Campanella, Laura Colombaioni, Edoardo Benedetti, Agostino Di Ciaula, Lisa Ghezzi, Massimo Onor, Massimo D’Orazio, Roberto Giannecchini, Riccardo Petrini, Emilia Bramanti

**Affiliations:** 1 National Research Council of Italy, C.N.R., Institute of Chemistry of Organo Metallic Compounds-ICCOM, 56124 Pisa, Italy; beatrice.campanella@pi.iccom.cnr.it (B.C.); onor@pi.iccom.cnr.it (M.O.); 2 CNR Neuroscience Institute, Area della Ricerca CNR, 56124 Pisa, Italy; laura.colombaioni@in.cnr.it; 3 Hematology Unit, Department of Oncology, University of Pisa, 56126 Pisa, Italy; edobenedetti@gmail.com; 4 International Society of Doctors for Environment (ISDE), 52100 Arezzo, Italy; agostinodiciaula@tiscali.it; 5 Department of Earth Sciences, University of Pisa, 56126 Pisa, Italy; lisa.ghezzi@unipi.it (L.G.); massimo.dorazio@unipi.it (M.D.); roberto.giannecchini@unipi.it (R.G.); riccardo.petrini@unipi.it (R.P.)

**Keywords:** thallium, emerging contaminant, low dose effects, human health

## Abstract

A mini review of the toxicity of Thallium (Tl) at low doses is herein presented. Thallium has severe toxicity. Although its acute biological effects have been widely investigated and are well known, its biological effects on human health and in cell cultures at low doses (<100 μg/L) due, for example, to Tl chronic exposure via consumption of contaminated water or foods, have often been overlooked or underestimated. Relatively few papers have been published on this topic and are herein reviewed to provide a focused scientific opinion in the light of current worldwide regulatory issues.

## 1. Introduction

In the last decade, there has been a growing concern about thallium (Tl) as an emerging natural or anthropogenic contaminant in the world, and about its potential toxicity to humans through natural geological pathways [1,2]. Liu et al., by performing a preliminary health risk assessment of trace metals in river water, found that Tl is the most important contributor to non-carcinogenic health risk concerns [3]. Very recently, China included Tl in the screening and prioritization of chemical hazards for deriving human health from ambient water quality criteria [4].

Thallium levels up to 10 µg/L were found in the drinking water distributed in the area, likely due to the interaction between groundwater and thallium-bearing pyrite ores. Since 2010, indeed, several geologists of the University of Pisa have found that mine waters, reaching exceptional Tl concentrations up to 9000 µg/L, were (and still are) flowing in the abandoned mining area [5,6,7,8,9,10].

In September 2014, scientists from the University of Pisa informed the local authorities about Tl accumulation in the tap water distribution system. The assessment of human contamination in urine and hair of the interested population has been previously reported [11].

Beside important works that review Tl toxicity at high concentrations (e.g., acute intoxication) [1,12,13], the aim of this communication is to report and discuss the most recent studies on Tl toxicity at low doses.

## 2. Are Low Doses of Thallium Toxic?

Thallium has two principal oxidation states, Tl(I) and Tl(III), both of which are considered highly toxic to living organisms [14]. Tl(I) is expected to be the dominant species in aqueous solution at equilibrium with atmospheric oxygen and in the absence of complexing agents [15]. Thallium(I) as Tl^+^ rapidly distributes in the various body compartments competing with K^+^ [13], generally reaching all cells in the absence of any barriers. The high-affinity uptake of Tl^+^ by human neurons and myocardial cells is clearly demonstrated by its employment in diagnostics and medical imaging [16,17].

While Tl effects in acute intoxication are well known [18], as well as those on biological systems at high concentrations [19,20,21,22,23,24], the effects of Tl at low doses and for short exposure periods on cell cultures have only recently been evaluated [25,26].

Chou et al. found that in the HEK293 cell line treated with 5, 10, and 20 mg/L Tl^+^ for 24 h, the ribosome synthesis decreased, thus resulting in a decreased protein synthesis, impaired ribosome biogenesis, and the blockage of cell cycle progression and apoptosis [24].

However, even a single 48 h exposure to Tl^+^ doses more than 50 times lower (1, 10, and 100 μg/L) have been found to have significant effects on neuronal growth rate and morphology [25,26]. An early-onset mitochondrial dysfunction appears after Tl^+^ exposure, which is associated with signs of cellular deregulation such as neurite shortening, loss of substrate adhesion, and increase of cytoplasmic calcium. The dose-dependent alteration of mitochondrial ROS (mtROS) levels and transmembrane mitochondrial potential (ΔΨm) have been also observed at very low Tl concentrations (1 μg/L). The contents of ethanol and lactate are significantly and dose-dependently altered after Tl exposure [25,26].

All these results are consistent with significant alterations in energy metabolism, and they highlight that mitochondria are a key sub-cellular target of Tl neurotoxic action.

More importantly, several authors have described significant correlations between the recorded effects on human health and low concentrations of Tl in human urine, serum, and blood. Table 1 summarizes an update list of papers published on this topic.

All these studies highlight that Tl exposure, even at very low concentrations, represents a threat to human health. Recently, a systematic risk characterization related to the long-term dietary exposure of the population to potentially toxic elements showed that the cumulative toxicity was mainly driven by Tl and vanadium [40].

These findings are not surprising, considering that Tl^+^ rapidly distributes in the various body compartments competing with K^+^ [13], as confirmed by its diagnostic employment in imaging techniques [16,17]. The evidence identifies mitochondria as the main target of Tl, but the biological mechanisms behind its action need to be further explored.

## 3. A Plea for Regulation Authorities

Thallium is an emerging pollutant. Its presence together with other very toxic, heavy metals close to mines, incinerators, and industries is proven. The widespread use of Tl, and its subsequent release into the environment has led to an increase in Tl levels in several environmental compartments and ecosystems and trophic chains, increasing its exposure to humans and other living organisms.

Publications in the last ten years on the effects on human health of Tl at doses by far below the US-EPA maximum contaminant level in drinking water (2 μg/L) have increased exponentially. U.S. EPA has the goal of lowering the maximum contaminant level of Tl in drinking water to 0.5 μg/L. However, ten years later, the MCL is still at 2, and no specific Italian or European regulation exists.

## 4. Conclusions

Further studies are certainly needed in order to fully explore the effects of Tl, at low concentrations, on human health. However, the available evidence cannot be disregarded. It should be unethical to ignore the most recent evidence waiting for a possible “*a posteriori*” demonstration of damages in the presence of a present and potentially manageable risk for public health.

Thus, data summarized in this minireview should encourage the regulation authorities to carefully and rapidly review the concentration limits of Tl in the various environmental compartments (biota, air, water, and land and aquatic sediments).

## Figures and Tables

**Table 1:** Papers published, which describe the effects on human health of low concentrations of Tl in urine/serum/blood.

Subjects	[Tl] (μg/L or μg/g Creatinine) in Urine/Serum/Blood	Summary of Health Effects	Ref.
n = 3800; age 6–60 y.o.	Geometric mean 0.176 μg/L (range 0.154–0.192) (urine)	Direct correlation with waist circumference and body mass index.	[27]
n = 1587 adults	0.15 (median) μg/L (range 0.11–0.21) (urine)	Impaired thyroid function (decrease of total thyroxine values, *P* < 0.05).	[28]
n = 55; age 5–16 y.o.	0.104 ± 0.083 μg/g creatinine (mean ± SD) (urine)	Positive correlation with Autism Spectrum Disorders (ASD).	[29]
n = 512; age 12–16 y.o.	0.27 μg/g creatinine (mean) (urine)	Positive correlation with estimated glomerular filtration rate.	[30]
n = 235 mothers and n = 241 neonates	Maternal blood 0.028 μg/L; cord blood = 0.017 μg/L	Placenta transports about 50% Tl from mother to fetus.	[31]
n = 816 (pregnant women)	Tl > 0.78 μg/g creatinine (0.02 < range < 8.15 μg/g) (urine)	Low birth weight.	[32]
n = 67	Average 0.17 µg/g creatinine; 25th and 75th percentiles normalized to the median of the control values: 0.10/0.20 (urine)	Positive correlation with ASD.	[33]
n = 53	Mean 0.510 μg/L (range 0.056–1.401) (urine)	Positive significant correlation (*P* < 0.01) with urinary 8-hydroxy-2′- deoxyguanosine (8-OHdG), a biomarker of DNA oxidative stress.	[34]
n = 750 (pregnant woman)	3rd trimester: 0.13 µg/g creatinine (range 0.092–0.18)	Thallium is associated with increased scyllo-inositol, acetate, formate, carnitine, and decreased dimethylamine and N-acetylated metabolites.	[35]
n = 3080 (pregnant woman and child until 2 years)	Median (P_25_–P_75_) of Tl levels in umbilical cord serum: 1st trimester: 0.062 μg/L (range 0.051–0.077); 2nd trimester: 0.060 μg/L (range 0.051–0.075); 3rd trimester: 0.04 (range 0.034–0.044) μg/L	Prenatal Tl exposure was associated with the reduction in infantile weight-for-age and height-for-age up to the age of 2 years and that these impacts might differ by gender.	[36]
n = 3013 women	Median = 0.062 μg/L (0.011–0.232 μg/L (serum)	Risk of gestational diabetes mellitus.	[37]
n = 1243 workers in coke-oven plant	0.58 μg/L (range 0.37–0.86 μg/L); 0.41 μg/g creatinine (range 0.27–0.64) (urine)	Deleterious effect on lung function, likely enhanced by tobacco smoking.	[38]
n = 746 pregnant women	Geometric mean value (maternal urine): 0.34 μg/L (1st trimester); 0.36 μg/L (2nd trimester); 0.34 μg/L (3rd trimester)	Negative association with blood leukocyte mtDNAcn in newborns shows that mitochondria is the target of thallium toxicity in early pregnancy.	[39]

CI = confidence interval; SD = standard deviation; DL = detection limit.
